# A novel quick contrast sensitivity function test in Chinese adults with myopia and its related parameters

**DOI:** 10.1007/s00417-023-06010-7

**Published:** 2023-02-21

**Authors:** Yuhao Ye, Aruma Aruma, Wuxiao Zhao, Zhong-Lin Lu, Xingtao Zhou, Jing Zhao

**Affiliations:** 1 Department of Ophthalmology and Optometry, Eye & ENT Hospital, Fudan University, Shanghai, China; 2 NHC Key Laboratory of Myopia (Fudan University), Key Laboratory of Myopia, Chinese Academy of Medical Sciences, 83 Fenyang Road, Shanghai, 200031 China; 3 Shanghai Research Center of Ophthalmology and Optometry, Shanghai, China; 4 Shanghai Engineering Research Center of Laser and Autostereoscopic 3D for Vision Care (20DZ2255000), Shanghai, China; 5 Division of Arts and Sciences, NYU Shanghai, Shanghai, China; Center for Neural Science and Department of Psychology, New York University, New York, NY USA; 6 NYU-ECNU Institute of Brain and Cognitive Science, NYU Shanghai, Shanghai, China

**Keywords:** Contrast sensitivity function, qCSF test, AULCSF, Spatial frequency, Myopia

## Abstract

**Purpose:**

This study is to investigate the contrast sensitivity function (CSF) using the quick CSF (qCSF) test in Chinese adults with myopia.

**Methods:**

This case series study included 320 myopic eyes of 160 patients (mean age 27.75 ± 5.99 years) who underwent a qCSF test for acuity, area under log CSF (AULCSF), and mean contrast sensitivity (CS) at 1.0, 1.5, 3.0, 6.0, 12.0, and 18.0 cycle per degree (cpd). Spherical equivalent, corrected-distant visual acuity (CDVA), and pupil size were recorded.

**Results:**

The spherical equivalent, CDVA (LogMAR), spherical refraction, cylindrical refraction, and the scotopic pupil size of the included eyes were − 6.30 ± 2.27 D (− 14.25 to − 0.88 D), 0 ± 0.02, − 5.74 ± 2.18 D, − 1.11 ± 0.86 D, and 6.77 ± 0.73 mm, respectively. The AULCSF and CSF acuity were 1.01 ± 0.21 and 18.45 ± 5.39 cpd, respectively. The mean CS (log units) at six different spatial frequencies were 1.25 ± 0.14, 1.29 ± 0.14, 1.25 ± 0.14, 0.98 ± 0.26, 0.45 ± 0.28, and 0.13 ± 0.17, respectively. A mixed effect model showed significant correlations between age and acuity, AULCSF, and CSF at 1.0, 12.0, and 18.0 cpd. Interocular CSF differences were correlated with the interocular difference of spherical equivalent, spherical refraction (at 1.0 cpd, 1.5 cpd), and cylindrical refraction (at 12.0 cpd, 18.0 cpd). The lower cylindrical refraction eye had higher CSF compared with the higher cylindrical refraction eye (0.48 ± 0.29 vs. 0.42 ± 0.27 at 12.0 cpd and 0.15 ± 0.19 vs. 0.12 ± 0.15 at 18.0 cpd).

**Conclusions:**

The age-related decrease in contrast sensitivity is at low and high spatial frequencies. Higher-degree myopia may show a decrease in CSF acuity. Low astigmatism was noted to affect the contrast sensitivity significantly.

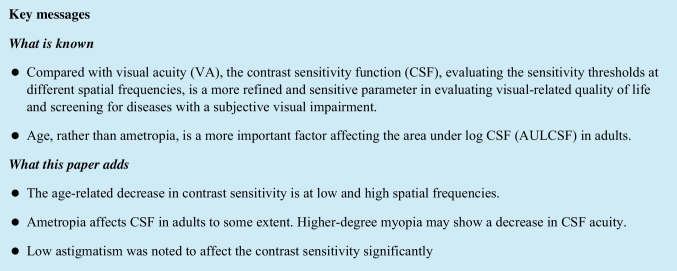



## Introduction

Contrast sensitivity function (CSF), evaluating the sensitivity thresholds at different spatial frequencies, is a more refined and sensitive parameter than visual acuity (VA) in evaluating visual-related quality of life and screening for diseases with a subjective visual impairment [1–4]. The introduction of the quick CSF (qCSF) test shortened the detection time, improved the sampling resolution, and increased repeatability, expanding its potential clinical application [1]. Previous studies have shown that qCSF test can be utilized to screen for age-related macular degeneration and [1, 2] multiple sclerosis [3], to detect mild to moderate changes in visual quality after refractive surgery [4], to classify patients with inherited retinal degeneration without obvious VA changes [5], to differentiate patients with amblyopia patients [6, 7] from those myopia [8], and to evaluate the effects of visual training in children with amblyopia. [9, 10]

Myopia is estimated to affect 50% of the world’s population by 2050 [11]. Myopia has been the prime focus of optometrists and ophthalmologists because it plays a crucial role in visual health and quality of life. A previous study suggested that patients with myopia show worse optical quality and visual function [12]. Traditional methods for measuring CSF are relatively time-consuming, and their low number of contrast levels greatly limits the range and resolution of the test grating stimuli. The novel qCSF test provides a more comprehensive evaluation of contrast sensitivity (CS) changes at a greater number of contrast and spatial frequency combinations. Age, rather than ametropia, has been reported as a more important factor affecting the area under log CSF (AULCSF) in adults [13]. There are a lot of paradoxical reports in literature on the association between CSF and age in individuals with myopia, with a lack of standardization of groups or the factors used for assessment. In clinical practice, the qCSF test results seem to highlight the differences between the two eyes of one patient with refractive correction. It is speculated that the refractive and biometric parameters as well as prescription of eyeglasses or contact lenses may affect qCSF readings. To better elucidate the relationship between qCSF readings and myopia, we postulated that research on myopes with further stratification of groups and the inclusion of additional possible factors affecting CSF distribution would be beneficial. This study assessed the qCSF of adults of different ages having different magnitudes of myopia with corrected VA. As qCSF is a quick test, we also postulated that the findings of this study could help assist in optometric prescription, and in visual function evaluation after refractive surgery, by comparing with a healthy control group.

## Methods

### Patients

The present study is a prospective cohort study, which included patients with myopia. The following were inclusion criteria: (i) patients aged ≥ 18 years; (ii) patients that had not used soft contact lenses for ≥ 2 weeks, or rigid gas permeable contact lenses for ≥ 4 weeks before the start of the study; (iii) patients with corrected-distant visual acuity (CDVA, LogMAR) ranging from − 0.1 to 0.1. The exclusion criteria were patients with history of cataract, glaucoma, or other eye diseases and/or a history of systemic diseases.

The study was approved by the ethics committee of Eye & ENT Hospital of Fudan University (2,020,107) and was conducted in accordance with the tenets of the Declaration of Helsinki. After obtaining written informed consent, patients were enrolled at the Eye & ENT Hospital of Fudan University, China, from November 2020 to July 2021.

**Fig. 1:**
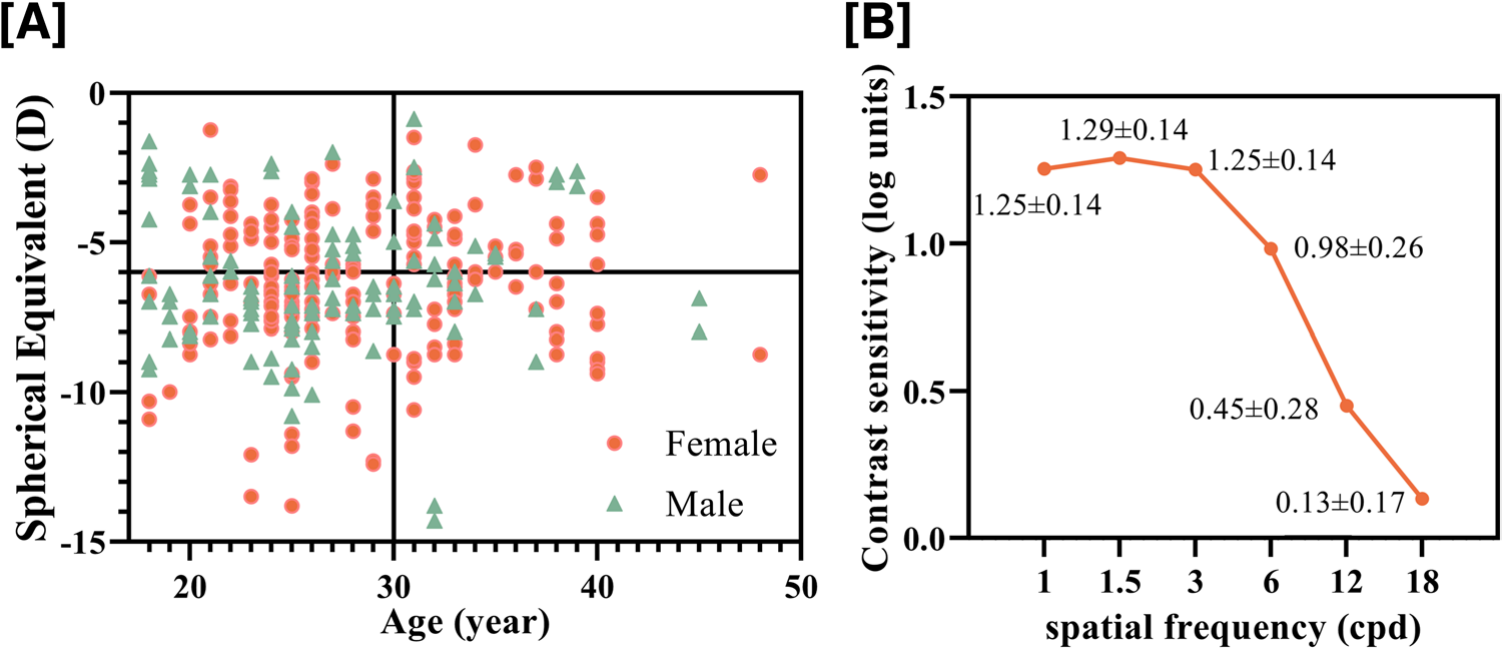
Distribution of age and refraction (**A**) and the qCSF (quick contrast sensitivity function) test results in all subjects (**B**)**A** The horizontal and vertical lines divide subjects into groups according to age (> or < 30 years) and spherical equivalent refraction (> or <  − 6.0 D)

This study included 320 myopic eyes of 160 cases (men:women = 57:103, mean age: 27.75 ± 5.99 [18, 48]). Table 1 and Fig. 1 represent characteristics of patients included in this study.

**Table 1:** Patient demographics and qCSF (quick contrast sensitivity function) test

Characteristic	Mean ± SD	Range
Age (years)	27.75 ± 5.99	(18, 48)
Gender (male/female)	57/103	
Axial length (mm)	26.17 ± 1.04	(23.49, 26.17)
PS (mm) (lamp off)	6.77 ± 0.73	(4.90, 8.80)
SR (D)	− 5.74 ± 2.18	(− 13.75, 0)
CR (D)	− 1.11 ± 0.86	(− 4.75, 0)
Spherical equivalent: SER (D)	− 6.30 ± 2.27	(− 14.25, − 0.88)
CDVA (LogMAR)	0 ± 0.02	(− 0.10, 0.10)
AULCSF	1.01 ± 0.21	(0.44, 1.50)
CSF acuity	18.45 ± 5.39	(7.00, 31.40)
1.0c/d	1.25 ± 0.14	(0.70, 1.62)
1.5c/d	1.29 ± 0.14	(0.31, 1.58)
3.0c/d	1.25 ± 0.14	(0, 0.67)
6.0c/d	0.98 ± 0.26	(0, 1.50)
12.0c/d	0.45 ± 0.28	(0, 1.14)
18.0c/d	0.13 ± 0.17	(0, 0.86)

Abbreviations: *PS* pupil size, *SR* spherical refraction, *CR*, cylindrical refraction, *SER* spherical equivalent, *CDVA* corrected-distant visual acuity, *AULCSF* area under log CSF

### Examinations

Spherical refraction (nearsightedness or farsightedness), cylindrical refraction (astigmatism), and CDVA measurements were performed by optometrists using a phoropter (RT-5100, Nidek Technologies, Japan), before and after the pupillary dilation. Pupil size (PS, lamp off) was measured using Nidek ARK-1(Nidek Technologies) under scotopic conditions. The qCSF test was conducted after the patients had corrected VA by wearing eyeglasses, according to the optometry results. Slit lamp examination and fundus examination were completed after pupillary dilation to exclude other eye diseases.

### qCSF test

**Fig. 2:**
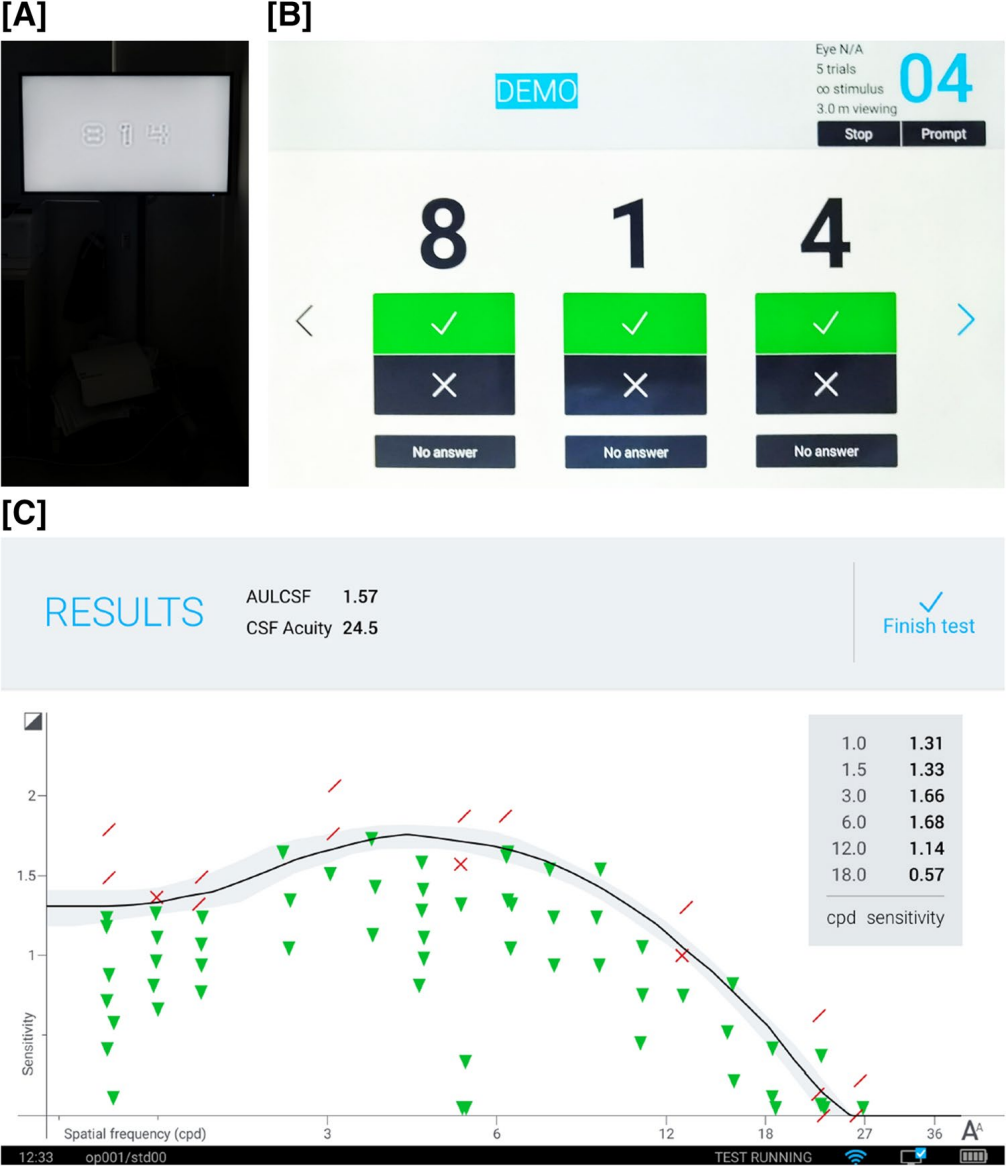
The interface of quick contrast sensitivity function test**A** Three filtered digits displayed on the screen as visual stimuli**B** The technician’s terminal displayed on the tablet**C** The qCSF test results. Green inverted triangle, correct answers; red cross fork, wrong answers; and the red slash, no answers

Each stimulus consisted of three numbers displayed on a NEC P403 monitor (Gension & Waltai Digital Video System Co., Ltd. China) with a display area of 116.84 × 77.89 cm, resolution of 1920 × 1080, maximum brightness of 700 cd/m^2^ and standard brightness 550 cd/m^2^, and contrast ratio of 4000:1. Sloan font was used for the numbers in the qCSF test (Fig. 2A). Previous research indicated that the 10-digit method qCSF test (stimuli shown as numbers from 0 to 9) showed a similar precision to that of the Psi method (stimuli shown as gratings or letters) and was effective, accurate, and appropriate for people not familiar to letters [14]. Participants viewed the stimuli horizontally at a distance of 3 m in a dark room through distance correction, via one eye, with the contralateral one covered. The other eye was then separately tested in a similar manner.

The screen showed 25 stimuli and 3 numbers, each at different spatial frequencies. The stimuli were generated using a Bayesian adaptive procedure, and the digits were presented in fovea. A computer directly depicted the CSF curve with estimation and analysis of four parameters: peak CS (CSmax), peak spatial frequency (SFmax), bandwidth β (the function’s full-width at half-maximum [in octaves]), and low contrast intercept δ (the truncation level at low spatial frequencies). The personalized stimuli were selected from a total of 2432 optotypes with 19 spatial frequencies and 128 contrast levels, depending on the participants’ response to the previous stimuli. At a sufficiently low contrast, the screen would appear uniform and unpatterned; at higher contrasts, the pattern would be visible [15, 16]. Patients were asked to read out the numbers on the screen, and the examiner immediately recorded their responses on a notepad as: “no answer,” “wrong answer,” or “correct answer” (Fig. 2B). The time taken to complete the test was similar between various groups (groupings shown below) in this study at around 3 min for each eye. The final results were presented as AULCSF, CSF acuity, and CS under six different spatial frequencies:1.0, 1.5, 3.0, 6.0, 12.0, and 18.0 cycle per degree (cpd) (Fig. 2C). The AULCSF describes the general spatial visual representation [17]. CSF acuity and the CS at different spatial frequencies correspond to the high frequency resolution state of the visual system under general and specific conditions, respectively [18].

### Statistical analysis

Statistical Package for Social Sciences (version 25.0, SPSS, Inc., Chicago, IL, USA) was used for data analysis. Mean ± standard deviation were used for data expression; the Shapiro–Wilk test was used to evaluate data normality, and a mixed effect model was used for multi-level correlation analysis, in which the analyzed correlated parameters were independent of each other and the effect of inter-eye correlation was removed. Patients were grouped in the following two ways for the purpose of the study. First, the patients were categorized according to possible correlated factors: (i) age > or < 30 years (the median age of the two groups were 25 and 33 years, respectively); (ii) cylindrical refraction > or <  − 2.0 DC; (iii) spherical refraction > or <  − 6.0 D; and spherical equivalent refraction (SER, SER = cylindrical refraction/2 + spherical refraction) > or <  − 6.0 D. Second, eyes with higher values of spherical equivalent (closer to emmetropia), spherical refraction (lower-degree myopia), or cylindrical refraction (lower astigmatism) compared to the contralateral eye in each patient were placed in one group, and eyes with lower corresponding parameters were enrolled in another group. Age of 30 years was chosen as the demarcation point, because it serves to delineate young adults from middle-aged adults. Furthermore, spherical refraction was included to better describe the impact of the degree of myopia on qCSF readings, because spherical equivalent included both spherical refraction and cylindrical refraction. Spearman correlation analysis was performed to analyze correlation between differences in parameters of two eyes based on qCSF values. A generalized estimation equation test was performed to analyze between-group differences, interocular differences, and correlated two-eye data in normally distributed data, in which the analyzed correlated parameters were independent of each other and the effect of inter-eye correlation was removed. The correlation was analyzed between the differences of the correlated parameters and the interocular difference of CSF to elucidate the impact of these correlated parameters on qCSF readings. The Wilcoxon signed-rank test was used for non-normally distributed data. A *p* value of < 0.05 indicated statistical significance.

## Results

### Correlation analysis

**Fig. 3:**
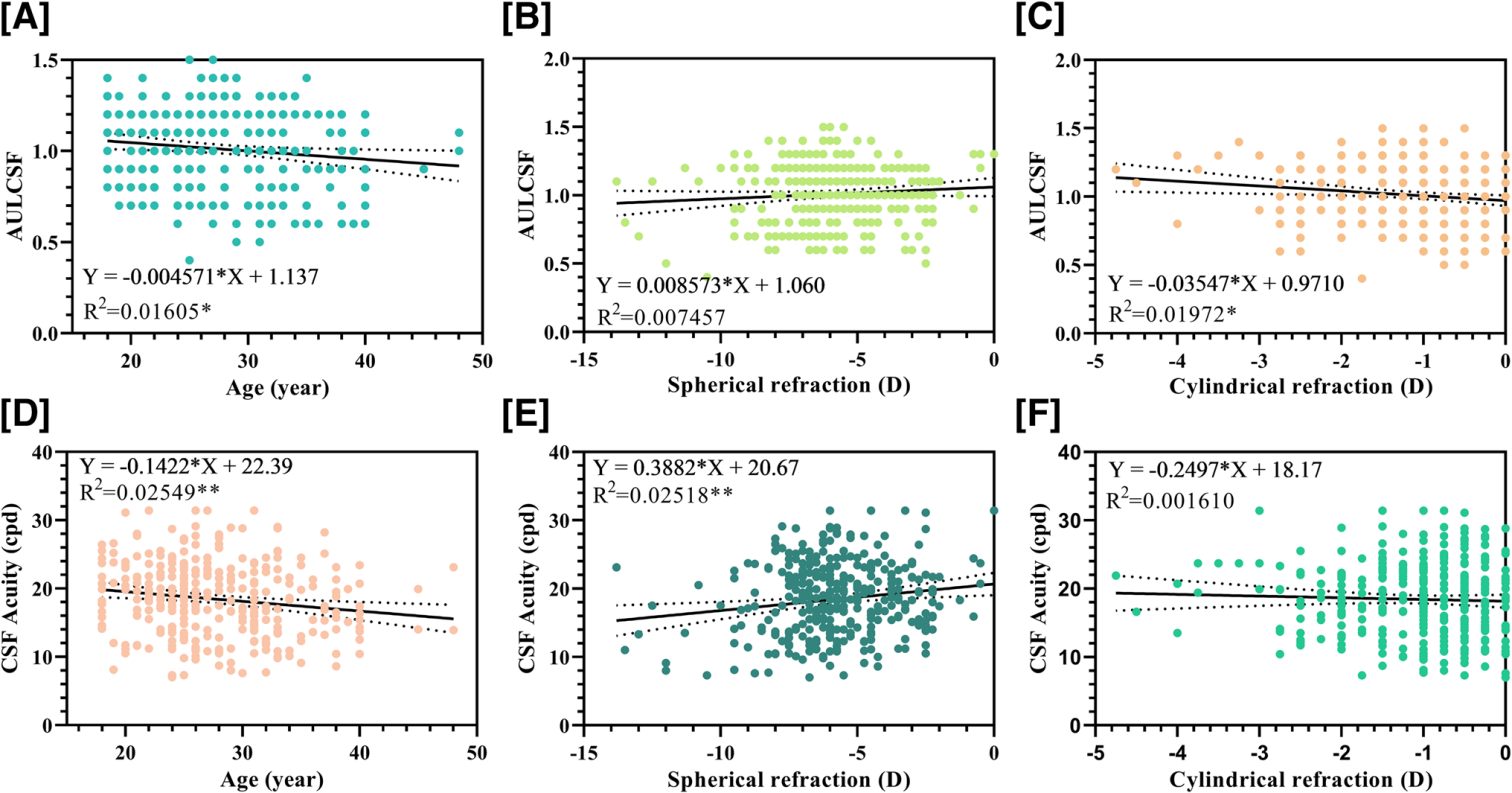
Correlations between qCSF parameters (AULCSF and CSF acuity) and age, spherical refraction (SR), or cylindrical refraction (CR). Simple linear regressions between AULCSF and CSF acuity and **A, D** age, **B, E** SR, and **C, F** CR. Solid lines illustrated the line of linear regressions and dotted line illustrated the confidential intervals. Dashed lines illustrated the lines of 95% confidential intervals. qCSF, quick contrast sensitivity function; AULCSF, area under log CSF (**P < 0.01, *P < 0.05)

Table 2 and Fig. 3 show CSF-correlated parameters and the corresponding coefficients. Age was significantly negatively correlated with AULCSF, CSF acuity, and CS at 1.0, 12.0, and 18.0 cpd. Spherical refraction was significantly positively correlated with CSF acuity. Cylindrical refraction was significantly positively correlated with AULCSF and CS at 1.5 cpd, while PS was significantly negatively correlated with CS at 1.0, 1.5, and 3.0 cpd.

**Table 2:** Association between qCSF (quick contrast sensitivity function) parameters with other factors analyzed by a mixed effects model

qCSF	Factors	*R* (fixed effect)	*P*
AULCSF	Age	− 0.928	0.039
	CR	0.337	0.010
CSF acuity	Age	− 0.839	0.003
	SR	0.497	0.003
1.0 c/d	Age	− 0.569	0.025
	PS	− 0.902	0.014
1.5 c/d	CR	0.177	0.018
	PS	− 0.909	0.004
3.0 c/d	PS	− 0.982	0.003
6.0 c/d	-	-	-
12.0 c/d	Age	− 0.978	0.025
18.0 c/d	Age	− 0.978	< 0.001

*CR* cylindrical refraction, *SR* spherical refraction, *PS* pupil size (lamp off), *AULCSF* area under log CSF

### Group comparison

**Fig. 4:**
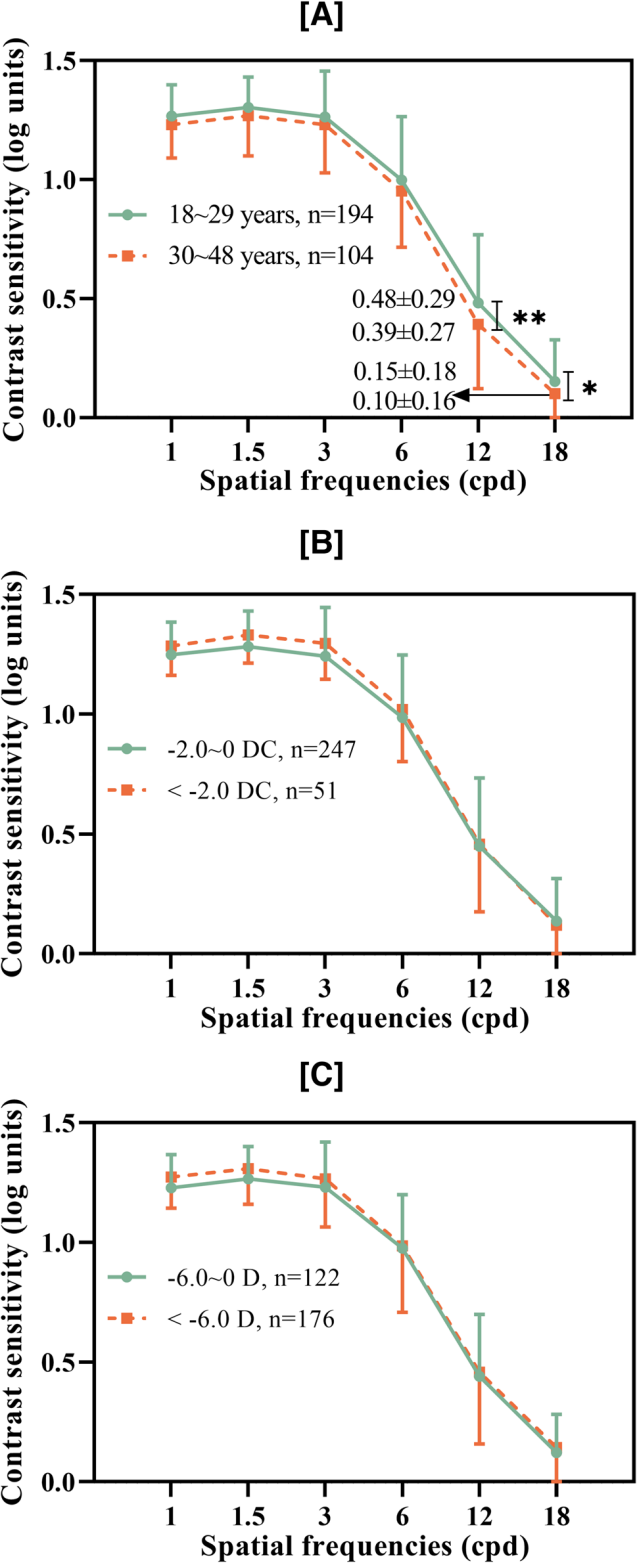
qCSF test of 320 eyes with myopia according to various grouping parameters**A** AULCSF, CSF acuity, and mean CS at each spatial frequency in stratified age groups**B** AULCSF, CSF acuity, and mean CS at each spatial frequency in stratified cylindrical refraction groups**C** AULCSF, CSF acuity, and mean CS at each spatial frequency in stratified spherical equivalent refraction groups. qCSF, quick contrast sensitivity function; AULCSF, area under log CSF; SR, spherical refraction; CR, cylindrical refraction; D, diopter (**P < 0.01, *P < 0.05)

The AULCSF, CSF acuity, and contrast sensitivity at high spatial frequencies of subjects < 30 years were significantly higher than those of patients aged > 30 years. However, no differences were found between patients grouped according to cylindrical refraction or SER (Table 3 and Fig. 4).

**Table 3:** Comparison of qCSF (quick contrast sensitivity function) readings in different groups in generalized estimating equations

Groups		AULCSF	CSF acuity	CSF (1.0 c/d)	CSF (1.5 c/d)	CSF (3.0 c/d)	CSF (6.0 c/d)	CSF (12.0 c/d)	CSF (18.0 c/d)
Ages (years)	18 ~ 29	**1.03 ± 0.21**	**19.07 ± 5.40**	1.26 ± 0.13	1.30 ± 0.13	1.26 ± 0.19	1.00 ± 0.27	**0.48 ± 0.24**	**0.15 ± 0.18**
	30 ~ 48	**0.97 ± 0.21**	**17.35 ± 5.05**	1.23 ± 0.13	1.27 ± 0.17	1.23 ± 0.20	0.95 ± 0.24	**0.39 ± 0.27**	**0.10 ± 0.16**
SR(D)	− 6.0 ~ 0	1.01 ± 0.19	18.83 ± 5.03	1.24 ± 0.19	1.28 ± 0.14	1.24 ± 0.19	0.95 ± 0.25	0.46 ± 0.26	0.13 ± 0.17
	< − 6.0	1.00 ± 0.23	18.05 ± 5.60	1.27 ± 0.13	1.30 ± 0.15	1.30 ± 0.15	1.01 ± 0.26	0.44 ± 0.30	0.14 ± 0.17
CR(D)	− 2.0 ~ 0	1.00 ± 0.21	18.46 ± 5.48	1.24 ± 0.14	1.28 ± 0.15	1.24 ± 0.20	0.99 ± 0.26	0.45 ± 0.28	0.14 ± 0.18
	< − 2.0	1.35 ± 0.20	18.41 ± 4.70	1.28 ± 0.12	1.33 ± 0.12	1.30 ± 0.15	1.02 ± 0.22	0.45 ± 0.28	0.12 ± 0.15
SER(D)	− 6.0 ~ 0	0.99 ± 0.18	18.60 ± 5.11	1.23 ± 0.14	1.27 ± 0.14	1.23 ± 0.19	0.98 ± 0.22	0.44 ± 0.26	0.12 ± 0.16
	< − 6.0	1.01 ± 0.23	18.34 ± 5.49	1.27 ± 0.13	1.31 ± 0.15	1.27 ± 0.20	0.99 ± 0.28	0.45 ± 0.30	0.14 ± 0.18

*CR* cylindrical refraction, *D* diopter, *SER* spherical equivalent, *AULCSF* area under log CSF

Values with statistical significance are shown in bold

### Interocular comparison

**Fig. 5:**
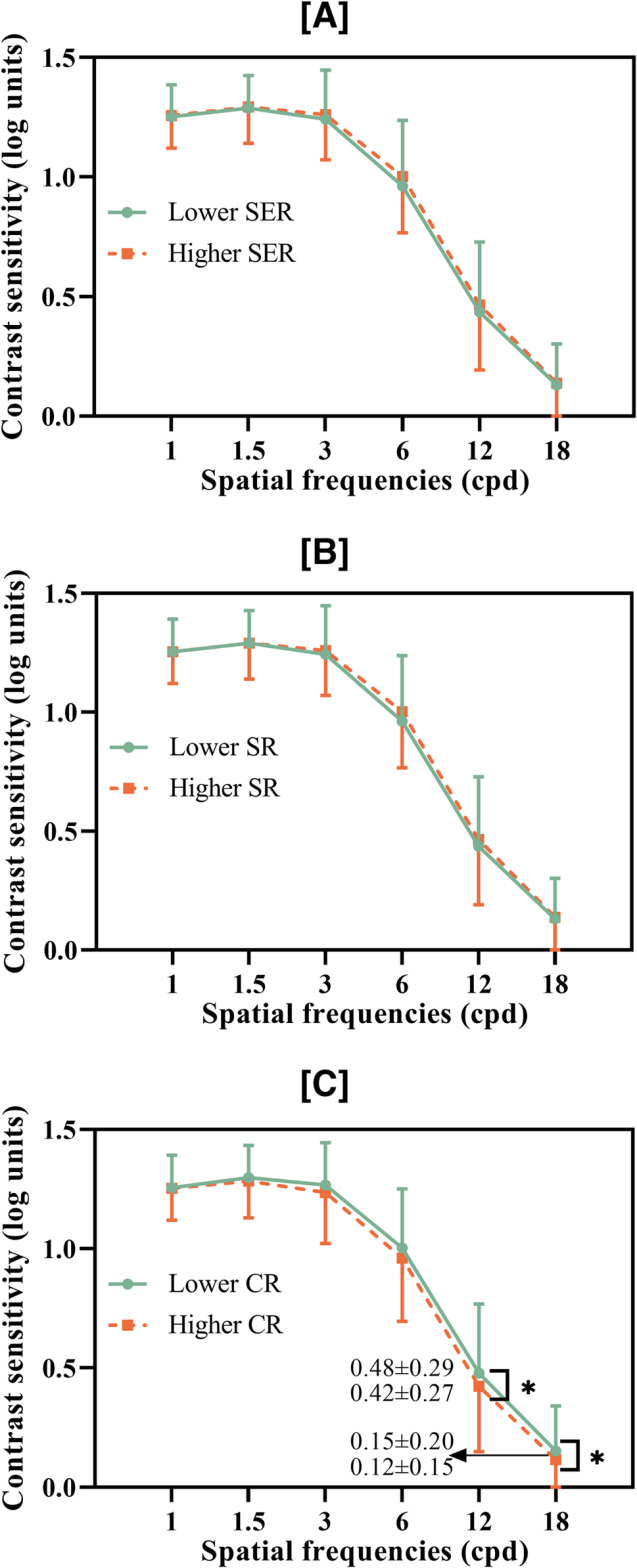
qCSF test of 320 eyes with myopia in groups stratified by interocular SER, SR, or CR differences**A** AULCSF, CSF acuity, and mean CS at each spatial frequency between groups stratified by interocular SER differences**B** AULCSF, CSF acuity, and mean CS at each spatial frequency between groups stratified by interocular SE differences**C** AULCSF, CSF acuity, and mean CS at each spatial frequency between groups stratified by interocular CR differences. qCSF, quick contrast sensitivity function; AULCSF, area under log CSF; SR, spherical refraction; CR, cylindrical refraction; D, diopter (**P < 0.01, *P < 0.05)

Table 4 shows the correlations between the binocular refractive difference and qCSF parameters. There was no significant correlation between age and interocular CS difference. The spherical refraction difference was significantly correlated to CS (at 1.0 and 1.5 cpd), the cylindrical refraction difference significantly correlated to contrast sensitivity (12.0 and 18.0 cpd), and the spherical equivalent difference was significantly correlated to CSF acuity and contrast sensitivity (at 1.0 and 1.5 cpd). There were 51 eyes in the higher-cylindrical refraction group and 34 eyes in the lower-cylindrical refraction group with cylindrical refractions between − 0.25 and 0.50 DC. Lower contrast sensitivity at high spatial frequency and CSF acuity were observed in higher cylindrical refraction groups compared with lower cylindrical refraction groups. However, there were no significant differences between groups in other qCSF parameters (Fig. 5C).

**Table 4:** Association between binocular difference of qCSF (quick contrast sensitivity function) readings with such of other factors in Spearman correlation analysis

qCSF parameters	Age		ΔSR		ΔCR		ΔSER	
	*r*	*P*	*r*	*P*	*r*	*P*	*r*	*P*
ΔAULCSF	− 0.107	0.179	0.084	0.291	0.056	0.487	0.099	0.217
ΔCSF acuity	− 0.119	0.135	0.152	0.055	0.127	0.111	**0.184**	**0.020**
Δ1.0 c/d	0.080	0.335	**0.168**	**0.041**	0.066	0.425	**0.185**	**0.024**
Δ1.5 c/d	0.027	0.745	**0.176**	**0.032**	0.101	0.219	**0.201**	**0.014**
Δ3.0 c/d	− 0.015	0.853	0.124	0.133	0.113	0.169	0.150	0.069
Δ6.0 c/d	− 0.029	0.731	0.074	0.370	0.093	0.262	0.095	0.252
Δ12.0 c/d	− 0.146	0.078	0.054	0.518	**0.243**	**0.003**	0.104	0.208
Δ18.0 c/d	− 0.138	0.094	− 0.044	0.592	**0.328**	**0.000**	0.021	0.797

*SR* spherical refraction, *CR* cylindrical refraction, *SER* spherical equivalent, *AULCSF* area under log CSF

Values with statistical significance are shown in bold

## Discussion

In the present study, a qCSF test was performed on adults with myopia that had corrected VA. The distribution pattern of qCSF at different spatial frequencies and the possible influencing factors behind it were identified.

A strong relationship between age and AULCSF has been previously reported. [13] The findings of the present study are in concordance with those of the previous study and further extended the specific aspects of the relationship between age and CS at high spatial frequencies. Previous studies have shown decreased CS at low to moderate spatial frequencies (of 1.5, 3.0, and 6.0 cpd) in patients with maculopathy having normal VA^2^, while in patients with inherited retinal degeneration having decreased VA, it was more likely to be affected at high spatial frequencies. [5] No retinopathy was found in the patients in this study; thus, age was still found to be a major factor affecting CS, even after eliminating the influence of refractive error at low and high spatial frequencies, pointing to the existence of the role of neural processes—other than retinal degeneration—in CSF.

The difference in CSF between patients with macular degeneration and an age-controlled group at low luminance was less than that observed under standard luminance, [19] and the CSF under photopic condition in post-laser-assisted in situ keratomileusis patients was significantly higher than that under moderate luminance. [4] Similarly, in this study, a larger PS (lamp off) may have reduced the CS at low spatial frequency. In this study, the analyzed correlated parameters (including age) were independent of each other, and the effect of inter-eye correlation was eliminated. In this case, the age-related change in PS might be interpreted as the factor of “age” exerting a larger impact on CS at high frequencies, resulting in lower correlation between PS and cCS at high spatial frequencies. We postulated that, in this study, aberration affected the qCSF tests results, rather than the age-related artefact.

In contrast to previous reports that demonstrated no significant correlation between refractive error and CSF, [13] we found a positive correlation between CSF Acuity and spherical refraction. One possible explanation might be spectacles used for distance visual acuity correction caused the curve shifting under different spatial frequencies. The optical media used for correction of myopia media may reduce image size, modify light transmission, have imperfect transparencies, and impair CSF acuity and CS at low frequencies [20]. Regarding analysis of interocular differences, intriguingly, the large interocular difference in spherical refraction was not in agreement with the significant difference in CS at low spatial frequencies. Previous studies have shown a correlation between CSF and VA in a population with low VA population. [21] However, no significant correlation between CSF and VA was reported in a population with normal VA. [22] A possible explanation for the lack of significant differences in the analysis of interocular differences in CS with respect to interocular spherical refraction may be the concentrated CDVA distribution in the patients of this study and its good performance at low spatial frequencies, as mild retinal changes although not significant may have occurred, while significant retinal changes may exist in patients with amblyopia [20]. Therefore, it could be concluded that the spherical refraction may obscure the analysis of contrast sensitivity at low spatial frequencies; additionally, further research is required to exclude interfering factors, such as optical media for visual correction.

The interocular comparison suggested that eyes with lower cylindrical refraction tended to show higher CS at high spatial frequencies. This finding suggests the impact of interocular differences in cylindrical refraction on CS at high spatial frequencies. Therefore, a possible interference of image processing at different spatial frequencies cannot be ruled out; the image processing of interocular vision may be similar at low spatial frequencies but different at high spatial frequencies. The effect of cylindrical refraction on visual quality is certain. [12] Interestingly, this finding shows that wearing cylindrical refraction-corrected glasses had a “reversible effect” on CSF. There were 51 eyes in the higher-cylindrical refraction group with a cylindrical refraction between − 0.50 and − 0.25 D and only 34 eyes in the lower-cylindrical refraction group. It is possible that a high cylindrical refraction (> − 0.5 D) may not get corrected in daily optometric management, affecting visual function during imaging processing. This finding may indicate the requirement of a full-correction optometric prescription under CSF guidance for high-cylindrical refraction eyes. Previous studies using monocular and interocular comparisons found that interocular observation shifted the CSF vertically upward. [23] Additionally, improved qCSF results were also detected after perceptual learning, suggesting the role of existence of neural processing in CSF. [18] Further research is required to better understand these correlations.

In group comparisons (Fig. 3), refractive error (spherical refraction, cylindrical refraction and SER) was not significantly different between groups; the concentrated distribution of patients might be the explanation for this. Analyses with larger sample sizes and further stratified groups will help to account for the difference in CSF. Further study including more patients with low myopia and high or ultra-high myopia are needed to eliminate the relatively concentrated distribution of patients with moderate myopia.

In this study, considerable time had to be allotted for optometry and for prescription of eyeglasses. Therefore, additional studies are needed to compare the time taken to perform the tests for children and adults and to evaluate the characteristics of qCSF results without the influence of refractive correction. No feedback was provided (patients were not informed of whether their responses were correct or not) during the qCSF tests to reduce the time required for possible neurobiological learning, which may have interfered with our test results.

The endpoint of manifest refraction is when adding + 0.25 DS to both eyes, it results in a slight blur of the patient’s best VA. An addition of − 0.25 DS will result in little effects, or it causes the letters to look darker and smaller. We chose patients with a LogMAR VA ranging from − 0.1 to 1.0 for this study. This range was set to minimize the effect of corrected VA on qCSF test results, because patients with a large difference of LogMAR VA may provide vastly different qCSF test results, under available VA correction. This specific inclusion criteria of the range of LogMAR VA could have helped in controlling the variables.

This study has some limitations. First, peripheral CSF measurements were not included. A previous study found a similar CSF on the temporal and nasal sides, [24] and the highest CSF was found in the horizontal field.  [24] Second, the study is limited by the lack of information on aberrations. Previous studies have shown that comatic aberrations significantly impact CSF in normal eyes. [17] More broadly, research is also needed to determine the correlation between different types of aberrations and CSF in patients with myopia and the specific spatial frequency of the effects. Third, this study excluded participants with soft contact lens used in 2 weeks or rigid gas permeable contact lens used in 4 weeks prior to the all the examination. Furthermore, aniseikonia may have been present or present to a greater degree with the use of corrective eyeglasses. Additionally, in patients with higher myopia, a smaller retinal image size occurs owing to a decrease in VA and CS at high spatial frequencies caused by the eyeglasses’ lens power effect. Therefore, further studies are warranted that exclude contact lens users and include the factor of eyeglasses’ lens power effect. Fourth, we did not include patients with emmetropia or hyperopia as a control group; future studies including patients with different refraction characteristics may help to elucidate the qCSF distribution more accurately. Finally, sample selection bias was considered a major limitation in this study because we included only those patients who visited the doctor for ophthalmological examinations and advice. The qCSF results are estimated to be lower in those subjects than in the general population with myopia.

In conclusion, the age-related decrease in CS was observed at low and high spatial frequencies. Higher-degree myopia may show a decrease in CSF acuity. Low astigmatism was noted to affect the CS significantly. Our findings have important implications in correction of optometric prescription under CSF guidance.

## Data Availability

The datasets generated and/or analyzed during the current study are not publicly available due to funding requirement but are available from the corresponding author on reasonable request.
